# Y Shape Osteotomy in Ankylosing Spondylitis, a Prospective Case Series with Minimum 2 Year Follow-Up

**DOI:** 10.1371/journal.pone.0167792

**Published:** 2016-12-09

**Authors:** Wenhao Hu, Jiayi Yu, Huawei Liu, Xuesong Zhang, Yan Wang

**Affiliations:** 1 Department of Orthopedics, Chinese PLA General Hospital, Beijing, People’s Republic of China; 2 Department of Renal cancer and Melanoma, Peking University Cancer Hospital, Beijing, People’s Republic of China; Mayo Clinic Minnesota, UNITED STATES

## Abstract

The aim of the study is to evaluate the efficacy of a spinal osteotomy technique, Y shape osteotomy, for correcting kyphosis in AS patients planned preoperatively with computer software-assistance. 36 consecutive AS patients with thoracolumbar kyphosis were treated with one-stage posterior Y shape osteotomy and preoperative surgical planning was done with the aid of the Surgimap Spine. Radiological parameters of simulation and immediate postoperation were documented. Clinical and radiological results were evaluated in the preoperative, the early postoperative periods and during the last follow-up. The lumbar lordosis was found as 40.7 ± 4.1 degrees in the surgical planning and 49.7 ± 3.9 degrees postoperatively (p<0.01). PI-LL was 3.8± 0.9°in the simulation procedure and 6.6± 1.5°postoperatively (p<0.01). At the final follow-up, Global sagittal balance was restored and Both Oswestry Disability Index and Scoliosis Research Society scores improved largely. In conclusion, Y shape osteotomy is a safe and effective treatment option for AS patients with kyphosis deformity.

## Introduction

Ankylosing spondylitis (AS) is a chronic inflammatory disease that results in progressive ossification of the ligaments of the spine and major joints. Over time, the spine becomes stiff and the normal sagittal alignment is lost. The clinical results are kyphotic deformity and impairment of the ability to stand upright. Surgical correction of the kyphosis is necessary in many patients with AS, to improve the visual field, respiratory function, balance, sitting position, swallowing function, and ambulation.[1, 2]

Y shape osteotomy is a technique for spinal osteotomy, which is characterised by controlled anterior column opening, posterior column closing and middle column preserving as the hinge in the meantime.[3] This technique has been performed as an effective treatment option for kyphotic deformity in patients with AS[4] and severe rigid congenital kyphoscoliosis and Pott's kyphosis.[5]

To obtain satisfactory outcomes, as we know, preoperative surgical planning must be performed. If not, overcorrection or undercorrection may be encountered and due to sagittal balance insufficiency, major complications may occur (such as rod breakage and pseudarthrosis)[6–8]. Surgimap Spine (Nemaris Inc, New York, NY, USA) is a dedicated spine measurement and surgical planning software, and Authors have advocated the use of this platform based on its effective and clinically useful osteotomy simulations and surgical planning[6].

In the current study, the purpose is to evaluate the long-term outcomes for Y shape osteotomy in patients with ankylosing spondylitis.

## Materials and Methods

36 consecutive AS patients with thoracolumbar kyphosis were treated with one-stage posterior Y shape osteotomy at a single vertebral level at our hospital between June 2011 and July 2013. This study was conducted with approval from the Ethics Committee of our Hospital.Written informed consent was obtained from all participants. The individual in this manuscript has given written informed consent (as outlined in PLOS consent form) to publish these case details. There were 28 males and 8 females with an average age at the time of operation of 38 years (range 27–52 years). The general information of patients was shown in Table 1.All patients underwent follow-up for a minimum of 24 months.

**Table 1:** Patient demographics.

case	Age/Sex	Osteotomy	Operative time	Blood loss	Follow-up
		level	(min)	(ml)	(mon)
1	28/M	L2	240	400	33
2	44/M	L1	260	450	34
3	41/M	L2	300	600	26
4	36/F	L2	190	350	30
5	52/M	L3	210	380	28
6	30/M	L2	220	400	32
7	33/M	L2	200	420	24
8	27/F	L3	270	500	32
9	45/M	L1	200	450	34
10	38/M	L2	230	440	28
11	40/M	L2	240	470	33
12	46/M	L2	200	390	35
13	39/M	L2	190	400	27
14	38/F	L1	250	470	32
15	46/M	L2	280	530	33
16	42/M	L2	210	440	26
17	31/M	L1	200	400	30
18	35/M	L2	230	450	34
19	30/M	L2	230	420	35
20	42/M	L1	250	430	32
21	43/F	L2	300	590	26
22	34/F	L2	210	360	30
23	51/M	L3	210	380	28
24	31/M	L2	220	400	32
25	32/M	L2	200	470	26
26	28/F	L3	250	450	30
27	45/M	L1	220	500	34
28	38/M	L2	240	390	28
29	46/M	L2	230	470	33
30	40/M	L2	220	390	35
31	34/M	L2	190	420	28
32	43/F	L1	230	450	31
33	46/M	L2	280	530	33
34	42/F	L2	230	430	26
35	32/M	L1	210	400	33
36	34/M	L2	200	460	31

The diagnosis of AS with rigid thoracolumbar kyphosis was made by radiographic examination, laboratory tests, and clinical features according to New York standards.[9] The indication for surgery in all cases was inability to stand upright or lie flat and inability to look straight ahead, and intractable back pain owing to muscle strain. Patients with vertebral rotation, coronal deformity, severe deformity requiring two-level osteotomy were excluded. In addition, patients were also excluded if they have received surgical interventional treatment before.

**Fig 1:**
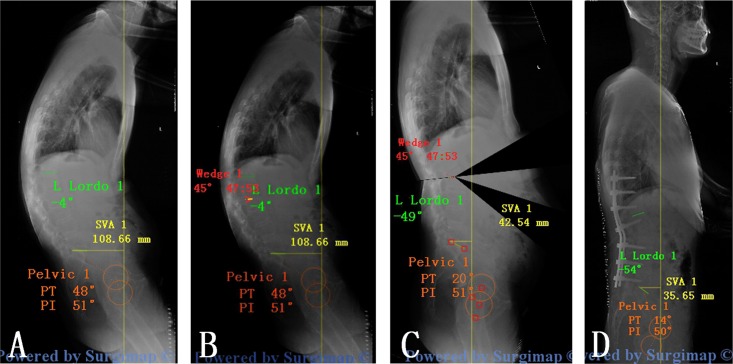
The simulation of Y shape osteotomy in surgimap for patients with AS. (A) Spino- pelvic parameters were measured and analyzed; (B) “Wedge Osteotomy” was applied at the posterior column of L2; (C) radiographic image after simulated osteotomy.

The patients were evaluated with anteroposterior and lateral full-length spinal radiographs, including the whole spine and pelvis. Preoperative lateral radiography was processed with the surgical planning software. Preoperative planning was executed in 3 phases: 1. Spino- pelvic parameters were measured and analyzed. lumbar lordosis (LL) (L1–S1), sagittal vertical axis(SVA)[10], pelvic tilt (PT), plvic incidence (PI) and sacral slope (SS)[11] were defined as the sagittal parameters and were measured on lateral radiographies. 2. The level of osteotomy was selected. When chosing the optimal osteotomy site, there are some factors we take into consideration: the greater correction with the lower neurological deficit risk, sufficient fixation distally while preserving motion segments. Osteotomy level was L1(n = 8), L2 (n = 24), L3(n = 4).3. Simulation of the osteotomy. This phase consists in applying a resection angle at the posterior column using the “Wedge Osteotomy” tool and graphically tracing the osteotomy directly on the radiographic image (Fig 1). The success of the correction according to the physiological limits described by Schwab et al. (SVA<50mm, PT<20mm and PI-LL<10mm)[11, 12].

The patients were all regularly followed with radiographs and clinical evaluations after 3, 12 and 24 months. Clinical improvement was assessed by Scoliosis Research Society-22 (SRS-22)[13] and The Oswestry Disability Index[14]. Operative time, blood loss, and general complications were recorded.

### Surgical procedures

**Fig 2:**
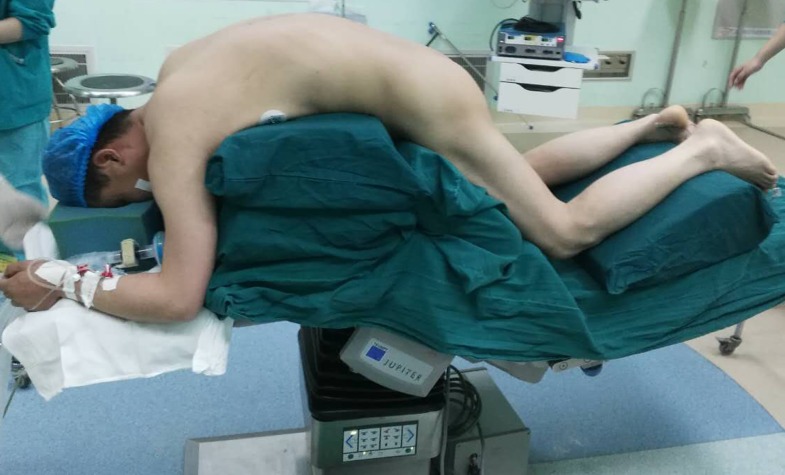
The patient is placed prone on a radiolucent operating table

In our center, all surgeries were performed with somatosensory-evoked potentials and transcranial motor-evoked potentials for neurophysiologic monitoring. Under general anesthesia, the patient was placed prone on the operating table depending on the degree of fixed kyphosis (Fig 2), a standard posterior middle incision was made at the pre-determined level. The spine was exposed by dissection lateral to the costotransverse joint at the thoracic level and the lumbar transverse process. Pedicle screws were then placed extending 3 levels above and 3 levels below the osteotomy site. Bleeding was controlled by electric cauterization and hemostatic gauze. The spinal canal was opened laterally, and the posterior elements including the spinous process, bilateral lamina, transverse process, and the adjacent facet joints at the vertebra to be osteotomised were removed as needed.

**Fig 3:**
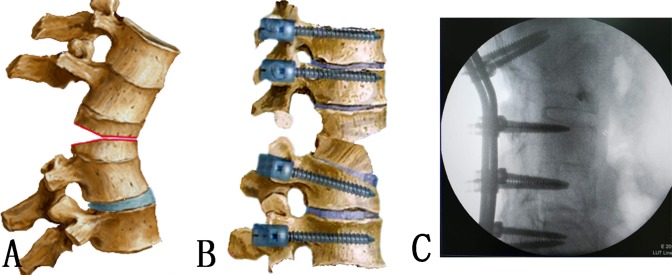
Y shape osteotomy. (A) ‘Y’ type osteotomy was achieved; (B) the posterior wedge space was closed with appropriate opening of the anterior column; (C) intra-operative imaging shows L1 Y shape osteotomy

The Y shape osteotomy was then performed (Fig 3). The pedicle probe and drill were used to create and enlarge pedicle holes of the target vertebra with both sides of the pedicles. Through the pedicle holes, the decancellous procedure was then performed within the posterior half of target column using rongeur and curette. The posterior cortical bone of the osteotomised vertebra was removed bilaterally with a Kerrison rongeur. A high-speed drill was used to make thinning of the anterior cortex and lateral walls of vertebral body, and osteoclasis of the anterior cortex and lateral walls then achieved using gentle manual extension when closing the posterior wedge space. In this procedure, an anterior opening wedge was created. The middle column was preserved as the hinge. The operating table and the position of the patient were adjusted for the correction. The technique is a ‘Y’ type osteotomy rather than V type osteotomy, which results in relative shortening of the posterior column, and appropriate opening of the anterior column, for adequate correction of the rigid kyphotic deformity.[15]A drainage tube was left in the surgical field, and the wound was closed in layers.

Postoperatively, the drainage tube was left until the output fell to <50 ml/24 h, usually after 3–5 days, and patients were instructed to use a plastic thoracolumbosacral orthosis during the first 3 months.

### Statistical analysis

Each variable is presented as the mean and standard deviation. Statistical analyses were performed using independent t-tests and paired t-tests (SPSS 17.0, SPSS Inc.). Normality was assumed, and a p value <0.05 was considered significant.

## Results

**Fig 4:**
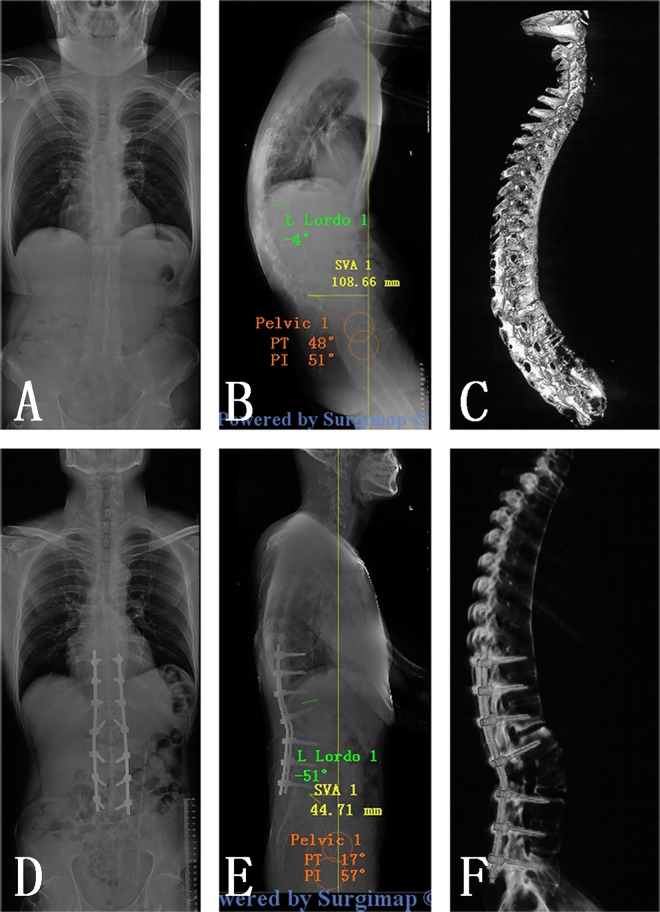
Pre- and post-operative radiological outcomes. (A, B, C) AP and lateral standing radiographs and sagittal CT scan of a 36-year-old man with thoracolumbar kyphosis secondary to ankylosing spondylitis; (D, E, F): Y shape osteotomy was performed at L2, and Two years of follow-up revealed the normal sagittal alignment was achieved.

Y shape osteotomy was performed in all patients according to preoperative surgical planning. The average operation time was 229 minutes (range 190–300 minutes) with a mean intraoperative blood loss of 441 ml (range 350–600 ml). The preoperative and last follow-up data of the 36 patients were summarized in Table 2.At the final follow-up, Global sagittal balance was restored in all patients. (Fig 4)

**Table 2:** The preoperative and last follow-up data of patients with AS.

Parameters	Preoperation	Postoperation	p-value
SVA(cm)	13.9 ± 3.9(9.5–20.6)	6.3 ± 1.3(4.1–7.9)	< 0.001
LL(°)	10.3 ± 3.9(4.0–17.0)	43.1 ± 3.8(38.0–50.0)	< 0.001
PT(°)	39.7 ± 3.8(35.0–48.0)	20.8 ± 2.3(18.0–25.0)	< 0.001
PI-LL(°)	40.3 ± 4.3(34.0–47.0)	12.3 ± 2.9(8.0–16.0)	< 0.001
SS(°)	9.4 ± 2.1(8.0–13.0)	19.4 ± 4.1(14.0–25.0)	< 0.001
CBVA(°)	27.7 ± 4.4(20.0–33.0)	8.3 ± 2.2(5.0–12.0)	< 0.001
ODI(%)	66.2 ± 8.5(52.6–78.5)	18.9 ± 4.6(12.6–26.5)	< 0.001
SRS			
Function	2.3 ± 0.7 (1.3–3.4)	4.5 ± 0.4 (3.8–5.0)	< 0.001
Pain	2.2 ± 1.0 (1.4–4.8)	4.4 ± 0.5 (3.6–5.0)	< 0.001
Appearance	1.8 ± 0.5 (1.0–2.4)	4.4 ± 0.2 (4.0–4.8)	< 0.001
Mental	2.2 ± 0.8 (1.2–3.8)	4.4 ± 0.4 (3.6–5.0)	< 0.001
Satisfaction	1.6 ± 0.4 (1.0–2.2)	4.5 ± 0.4 (3.8–5.0)	< 0.001

No major acute complications such as death or complete paralysis occurred. Dural tear with transient cerebrospinal fluid leakage was encountered in 1 case. These tears were covered intraoperatively by muscle, and lumbar drainage was placed and removed after 8 days. Bed rest was required during this period. The patient recovered without further complications and was discharged 11 days after surgery. There was 1 case of postoperative numbness in the left lower extremity that resolved within 8 weeks.

## Discussion

Ankylosing spondylitis is often associated with severe kyphotic deformity in the later stage. In sagittal imbalance conditions, muscle fatigue and low back pain are frequently encountered.

From a biomechanical perspective, the ideal method to restore the sagittal balance is shifting the center of gravity of the trunk over the hip axis.[16] Smith-Peterson osteotomy(SPO)[17], pedicle subtraction osteotomy(PSO),[18] vertebral column resection(VCR)[19] have been applied for correcting thoracolumbar kyphosis. However, SPO, an opening wedge osteotomy, is mainly suitable for treatment of flexible kyphosis deformities[20] and it is not frequently presented with the rigid deformity such as AS. PSO, or transpedicular wedge osteotomy, has been the mainstream type of osteotomy for patients with AS until now.[21] It is a closing wedging osteotomy which hinges at anterior column of vertebral body[22]. During the correction procedure, the posterior and middle columns were shortened without lengthening of the anterior column.[23] The excessive shortening of the area variable for the cord can be dangerous with a PSO and authors have recommended limiting the correction to 30–40°.[20] VCR is the most powerful tool for correction of spinal deformity, However, it is restricted owing to its technical difficulty and potential for complications.[24]

Y shape osteotomy is a spinal osteotomy as a combination of several osteotomy techniques including the eggshell technique, SPO, PSO and VCR. The key points in relation to the Y shape osteotomy are to remove the relative small amount of posterior half of the osteotomy column and preserve as much as possible of the middle column as the hinge, which serves as the correction ‘leverage’ to provide greater stability and better fusion during correction procedure. Opening of the anterior column results in larger correction angle and decreasing the need for shortening of the posterior column, which reduced the risk of sagittal translation and neurological sequelae.[25] Although opening of the anterior column incurs a theoretical increase in certain complications, we believe this is offset by the fact that the anterior wedge is smaller than other wedges created, for example, following an Smith-Petersen Osteotomy.[3] What’s more, osteoclasis of anterior cortex is suitable for correcting the rigid deformity in AS patients. As the eggshell technique, the order of vertebrae column decancellation in Y shape osteotomy was from inside to outside, which means it is not necessary to expose the segmental vessels in most cases, with a reduced risk of vascular complications occurring.[26] To our knowledge, our study is the largest series of AS treated by the Y shape osteotomy to data.

Proper restoration of the sagittal profile is critical to postoperative outcomes [27, 28] and surgical correction requires careful planning.[13, 16] However, a systematic approach to planning optimal correction is lacking. In one such study examining patients who underwent lumbar pedicle subtraction osteotomy (PSO), it was determined that 23% of realignment procedures failed.[7] Likewise, 22% of thoracic PSO patients were found to have poor postoperative spinopelvic alignment.[8] Surgimap Spine, a free computer program, has gained popularity recently among spine surgeons. With this software, it is either possible to evaluate the important spinopelvic parameters or to simulate the spinal osteotomy. In the current study, the software firstly provided an efficacious and accuracy analysis of sagittal alignment for each patient. Then, the Y shape osteotomy was simulated at the pre-determined level. When it was insufficient to restore the sagittal spinal parameters within the physiological limits during the preoperative planning process, VCR or two-level PSO was needed.

## Conclusion

The Y shape osteotomy is a safe and effective treatment option for AS patients with kyphosis deformity, this approach achieves satisfactory kyphosis correction and improvement in neurological function. Surgimap Spine as a dedicated spine measurement and surgical planning software provides a helpful method to analyze the spino-pelvic parameters and simulate the procedure of osteotomy.

## Supporting Information

S1 video Yshape osteotomy. (MP4)

## References

[1] Kubiak EN, Moskovich R, Errico TJ, Di Cesare PE. Orthopaedic management of ankylosing spondylitis. The Journal of the American Academy of Orthopaedic Surgeons. 2005;13(4):267–78. 16112983 10.5435/00124635-200507000-00006

[2] Glassman SD, Bridwell K, Dimar JR, Horton W, Berven S, Schwab F. The impact of positive sagittal balance in adult spinal deformity. Spine. 2005;30(18):2024–9. 16166889 10.1097/01.brs.0000179086.30449.96

[3] Mehdian H, Arun R, Aresti NA. V-Y vertebral body osteotomy for the treatment of fixed sagittal plane spinal deformity. The spine journal: official journal of the North American Spine Society. 2015;15(4):771–6. 25614149 10.1016/j.spinee.2015.01.014

[4] Zhang X, Zhang Z, Wang J, Lu M, Hu W, Wang Y, et al Vertebral column decancellation: a new spinal osteotomy technique for correcting rigid thoracolumbar kyphosis in patients with ankylosing spondylitis. The bone & joint journal. 2016;98-B(5):672–8. 27143740 10.1302/0301-620X.98B5.35726

[5] Wang Y, Zhang Y, Zhang X, Wang Z, Mao K, Chen C, et al Posterior-only multilevel modified vertebral column resection for extremely severe Pott's kyphotic deformity. European spine journal: official publication of the European Spine Society, the European Spinal Deformity Society, and the European Section of the Cervical Spine Research Society. 2009;18(10):1436–41. 10.1007/s00586-009-1067-9 PMC2899371 19526375

[6] Akbar M, Terran J, Ames CP, Lafage V, Schwab F. Use of Surgimap Spine in sagittal plane analysis, osteotomy planning, and correction calculation. Neurosurgery clinics of North America. 2013;24(2):163–72. 10.1016/j.nec.2012.12.007 23561555

[7] Schwab FJ, Patel A, Shaffrey CI, Smith JS, Farcy JP, Boachie-Adjei O, et al Sagittal realignment failures following pedicle subtraction osteotomy surgery: are we doing enough?: Clinical article. Journal of neurosurgery Spine. 2012;16(6):539–46. 10.3171/2012.2.SPINE11120 22462571

[8] Lafage V, Smith JS, Bess S, Schwab FJ, Ames CP, Klineberg E, et al Sagittal spino-pelvic alignment failures following three column thoracic osteotomy for adult spinal deformity. European spine journal: official publication of the European Spine Society, the European Spinal Deformity Society, and the European Section of the Cervical Spine Research Society. 2012;21(4):698–704. 10.1007/s00586-011-1967-3 PMC3326123 21837411

[9] Moll JM. New criteria for the diagnosis of ankylosing spondylitis. Scandinavian journal of rheumatology Supplement. 1987;65:12–24. 10.3109/03009748709102173 3317802

[10] Jackson RP, McManus AC. Radiographic analysis of sagittal plane alignment and balance in standing volunteers and patients with low back pain matched for age, sex, and size. A prospective controlled clinical study. Spine. 1994;19(14):1611–8. 7939998 10.1097/00007632-199407001-00010

[11] Legaye J, Duval-Beaupere G, Hecquet J, Marty C. Pelvic incidence: a fundamental pelvic parameter for three-dimensional regulation of spinal sagittal curves. European spine journal: official publication of the European Spine Society, the European Spinal Deformity Society, and the European Section of the Cervical Spine Research Society. 1998;7(2):99–103. 10.1007/s005860050038 PMC3611230 9629932

[12] Schwab F, Patel A, Ungar B, Farcy JP, Lafage V. Adult spinal deformity-postoperative standing imbalance: how much can you tolerate? An overview of key parameters in assessing alignment and planning corrective surgery. Spine. 2010;35(25):2224–31. 10.1097/BRS.0b013e3181ee6bd4 21102297

[13] Crawford CH 3rd, Glassman SD, Bridwell KH, Berven SH, Carreon LY. The minimum clinically important difference in SRS-22R total score, appearance, activity and pain domains after surgical treatment of adult spinal deformity. Spine. 2015;40(6):377–81. 10.1097/BRS.0000000000000761 25774463

[14] Kiaer T, Gehrchen M. Transpedicular closed wedge osteotomy in ankylosing spondylitis: results of surgical treatment and prospective outcome analysis. European spine journal: official publication of the European Spine Society, the European Spinal Deformity Society, and the European Section of the Cervical Spine Research Society. 2010;19(1):57–64. 10.1007/s00586-009-1104-8 PMC2899742 19662442

[15] Van Royen BJ, De Gast A. Lumbar osteotomy for correction of thoracolumbar kyphotic deformity in ankylosing spondylitis. A structured review of three methods of treatment. Annals of the rheumatic diseases. 1999;58(7):399–406. 10381482 10.1136/ard.58.7.399 PMC1752916

[16] Song K, Zheng G, Zhang Y, Zhang X, Mao K, Wang Y. A new method for calculating the exact angle required for spinal osteotomy. Spine. 2013;38(10):E616–20. 10.1097/BRS.0b013e31828b3299 23392416

[17] Smith-Petersen MN, Larson CB, Aufranc OE. Osteotomy of the spine for correction of flexion deformity in rheumatoid arthritis. Clinical orthopaedics and related research. 1969;66:6–9. 5357786

[18] Thomasen E. Vertebral osteotomy for correction of kyphosis in ankylosing spondylitis. Clinical orthopaedics and related research. 1985(194):142–52. 3978906

[19] Suk SI, Chung ER, Lee SM, Lee JH, Kim SS, Kim JH. Posterior vertebral column resection in fixed lumbosacral deformity. Spine. 2005;30(23):E703–10. 16319740 10.1097/01.brs.0000188190.90034.be

[20] Xi YM, Pan M, Wang ZJ, Zhang GQ, Shan R, Liu YJ, et al Correction of post-traumatic thoracolumbar kyphosis using pedicle subtraction osteotomy. European journal of orthopaedic surgery & traumatology: orthopedie traumatologie. 2013;23 Suppl 1:S59–66. 23412310 10.1007/s00590-013-1168-3

[21] Zheng GQ, Song K, Zhang YG, Wang Y, Huang P, Zhang XS, et al Two-level spinal osteotomy for severe thoracolumbar kyphosis in ankylosing spondylitis. Experience with 48 patients. Spine. 2014;39(13):1055–8. 10.1097/BRS.0000000000000346 24732843

[22] Qian BP, Wang XH, Qiu Y, Wang B, Zhu ZZ, Jiang J, et al The influence of closing-opening wedge osteotomy on sagittal balance in thoracolumbar kyphosis secondary to ankylosing spondylitis: a comparison with closing wedge osteotomy. Spine. 2012;37(16):1415–23. 10.1097/BRS.0b013e318250dc95 22391439

[23] Bridwell KH, Lewis SJ, Edwards C, Lenke LG, Iffrig TM, Berra A, et al Complications and outcomes of pedicle subtraction osteotomies for fixed sagittal imbalance. Spine. 2003;28(18):2093–101. 10.1097/01.BRS.0000090891.60232.70 14501920

[24] Suk KS, Kim KT, Lee SH, Kim JM. Significance of chin-brow vertical angle in correction of kyphotic deformity of ankylosing spondylitis patients. Spine. 2003;28(17):2001–5. 10.1097/01.BRS.0000083239.06023.78 12973148

[25] Murrey DB, Brigham CD, Kiebzak GM, Finger F, Chewning SJ. Transpedicular decompression and pedicle subtraction osteotomy (eggshell procedure): a retrospective review of 59 patients. Spine. 2002;27(21):2338–45. 10.1097/01.BRS.0000030853.62990.BC 12438981

[26] Arun R, Dabke HV, Mehdian H. Comparison of three types of lumbar osteotomy for ankylosing spondylitis: a case series and evolution of a safe technique for instrumented reduction. European spine journal: official publication of the European Spine Society, the European Spinal Deformity Society, and the European Section of the Cervical Spine Research Society. 2011;20(12):2252–60. 10.1007/s00586-011-1894-3 PMC3229721 21800034

[27] Blondel B, Schwab F, Ungar B, Smith J, Bridwell K, Glassman S, et al Impact of magnitude and percentage of global sagittal plane correction on health-related quality of life at 2-years follow-up. Neurosurgery. 2012;71(2):341–8; discussion 8. 10.1227/NEU.0b013e31825d20c0 22596038

[28] Smith JS, Klineberg E, Schwab F, Shaffrey CI, Moal B, Ames CP, et al Change in classification grade by the SRS-Schwab Adult Spinal Deformity Classification predicts impact on health-related quality of life measures: prospective analysis of operative and nonoperative treatment. Spine. 2013;38(19):1663–71. 10.1097/BRS.0b013e31829ec563 23759814

